# Identification and association of the single nucleotide polymorphisms in calpain3 (*CAPN3*) gene with carcass traits in chickens

**DOI:** 10.1186/1471-2156-10-10

**Published:** 2009-03-05

**Authors:** Zeng-Rong Zhang, Yi-Ping Liu, Yong-Gang Yao, Xiao-Song Jiang, Hua-Rui Du, Qing Zhu

**Affiliations:** 1 College of Animal Science and Technology, Sichuan Agriculture University, Ya'an, Sichuan 625014, PR China; 2 Key Laboratory of Animal Models and Human Disease Mechanisms, Kunming Institute of Zoology, Chinese Academy of Sciences, Kunming, Yunnan 650223, PR China; 3 Institute of Poultry Sciences, Sichuan Animal Science Academy, Chengdu, Sichuan 610066, PR China; 4 Breeding Center, Sichuan Dahen Poultry Breeding Company, Chengdu, Sichuan 610066, PR China

## Abstract

**Background:**

The aim of this study is to screen single nucleotide polymorphisms (SNP) of chicken *Calpain3 *(*CAPN3*) gene and to analyze the potential association between *CAPN3 *gene polymorphisms and carcass traits in chickens. We screened *CAPN3 *single nucleotide polymorphisms (SNP) in 307 meat-type quality chicken from 5 commercial pure lines (S01, S02, S03, S05, and D99) and 4 native breeds from Guangdong Province (Huiyang Huxu chicken and Qingyuan Ma chicken) and Sichuan Province (Caoke chicken and Shandi Black-bone chicken), China.

**Results:**

Two SNPs (11818T>A and 12814T>G) were detected by single strand conformation polymorphism (SSCP) method and were verified by DNA sequencing. Association analysis showed that the 12814T>G genotypes were significantly associated with body weight (BW), carcass weight (CW), breast muscle weight (BMW), and leg muscle weight (LMW). Haplotypes constructed on the two SNPs (H1, TG; H2, TT; H3, AG; and H4, AT) were associated with BW, CW (*P *< 0.05), eviscerated percentage (EP), semi-eviscerated percentage (SEP), breast muscle percentage (BMP), and leg muscle percentage (LMP) (*P *< 0.01). Diplotype H1H2 was dominant for BW, CW, and LMP, and H2H2 was dominant for EP, SEP, and BMP.

**Conclusion:**

We speculated that the *CAPN3 *gene was a major gene affecting chicken muscle growth and carcass traits or it was linked with the major gene(s). Diplotypes H1H2 and H2H2 might be advantageous for carcass traits.

## Background

The accumulation of muscle mass in the body is determined by the relative rates of muscle protein synthesis and degradation, namely, this process is closely related to the muscle protein turnover. Studies on the rate of muscle growth in layer and broiler chickens have shown that the rate of muscle growth are mainly determined by changes in the rate of muscle protein degradation, with little or no change in the rate of muscle protein synthesis [1].

Calpains, i.e. intracellular Ca^2+^-dependent cysteine proteases (EC 3.4.22.17), have long been demonstrated to be involved in muscle growth and development and are proenzymes that are regulated by Ca^2+ ^binding and autoproteolytic modification [2]. The calpains were discovered because the Z-disks in muscle strips incubated in a Ca^2+^-containing solution disappeared but with no other ultrastructurally detectable change [3]. Four calpain genes (*CAPN1*, *CAPN2*, *CAPN3*, and *CAPN1.5*) are expressed ubiquitously in chicken [4]. Study have further showed that there was less calpain activity, but more calpastatin, in broiler breast muscle [5]. Among the calpain family members, calpain 3 (Capn3, previously named p94) is particularly interesting and has two insertion sequences, IS1 and IS2, which are involved in the regulation of its function and activity [6]. *CAPN3 *was identified by positional cloning as the gene responsible for limb-girdle muscular dystrophy 2A (LGMD2A) [7,8], where it plays an important role in myofibrillar integrity [9]. Capn3 binds specifically to connectin (a striated muscle specific giant filamentous protein) at the N2 line [10], a region where proteolysis has been linked to post-mortem tenderization [11]. *CAPN3 *has received considerable attention in recent years because of its potential influence on muscle growth and its role in myofibrillar organization. However, much of the available information about this gene was taken from studies on humans, mice, and other mammalian species, and might not be directly applicable to poultry.

Calpain 3 is ubiquitously expressed in chicken [4] and is mainly located in skeletal muscle[10]. Compared to the other calpains, Capn3 activity is regulated by different mechanisms, as its activation and function as a muscle cytoskeleton regulator requires very low concentration of calcium and different interacting proteins [12]. We hypothesized that *CAPN3 *gene may be a major gene affecting chicken carcass traits.

In this study, we screened *CAPN3 *single nucleotide polymorphisms (SNP) in 307 meat-type quality chickens from 5 commercial pure lines (S01, S02, S03, S05, and D99) and 4 native breeds [Huiyang Huxu chicken (HH), Qingyuan Ma chicken (QY), Caoke chicken (CK) and Mountainous black-bone chicken (MB)] in China, with an intention to evaluate potential association between *CAPN3 *polymorphisms and the chicken carcass traits.

## Results

### SNPs of the Chicken CAPN3 Gene

According to UCSC Genome Browser on Chicken May 2006 Assembly  using *Gallus gallus Calpain3 *sequence (GenBank accession number XM_421157), chicken *CAPN3 *gene contains 24 exons. We chose exons 3, 4, 5, 8, 9, 10, 16, 17, and 19 for analyses based on a preliminary analysis of the BGI SNPs[13], which contain reads from the Broiler, Layer and Silkie chicken strains and are available at the UCSC Genome Browser. A PCR-based SSCP method was successfully developed for screening the nucleotide substitutions in these exons. Two SNPs, a T/A variant at position nt11818 (located in intron 8) and a T/G variant at nt12814 (located in exon 10), were identified in the chicken DNA sequence (GenBank Accession No: NC_006092.2, GI: 118136304). Variant 12814T>G was silent and did not cause the amino acid change.

### Genotype and Allele Frequencies in Different Chicken Populations

Additional file 1 showed the allele and genotype frequencies of the two SNPs in nine chicken populations. For SNP 11818T>A, the allele A was the dominant allele and presented the highest allele frequencies among all chicken populations (average = 58.67%). For SNP 12814T>G, the allele frequency of T was higher than that of G in populations S03 and S02, while in S01, D99, S05, QY, HH, and SD, the allele G was identified as the dominant one. The χ^2^-test for the genotype frequency showed that there were statistically significant differences among the nine chicken populations for 12814T>G (*P *< 0.01), whereas the difference for 11818T>A was not significant (*P *> 0.05). Furthermore, variant 11818T>A was significantly deviated from the Hardy-Weinberg equilibrium (HWE) in samples D99, S05, SD, and CK, whereas variant 12814T>G was not violated HWE.

### Association of CAPN3 SNPs with Carcass Traits

The results of the GLM analysis of association between the *CAPN3 *gene polymorphisms and carcass traits were summarized in Table 1. For 11818T>A, chickens with the AA genotype had significantly higher body weight (BW) than those chickens with the AT and TT genotypes, respectively (*P *< 0.05); chickens with the AA genotype also had significantly higher eviscerated percentage (EP) values compared to those of chickens with the AT genotype (*P *< 0.05), whereas the breast muscle percentage (BMP) (%) of chickens with the AA genotype was significantly higher than that of the TT genotype (*P *< 0.05). On contrast, leg muscle percentage (LMP) (%) of chickens with the AA genotype was significantly lower than that of the TT genotype (*P *< 0.01). No significant difference was detected for other carcass traits.

**Table 1:** Least square mean carcass traits, by genotype, of chicken *CAPN3 *for each locus

Trait	11818T>A			P*
		
	AA	AT	TT	
BW (g)	1615.80^a ^± 26.85	1568.22^b ^± 28.93	1578.44^b ^± 35.10	0.025
EP (%)	72.64^a ^± 0.27	72.09^b ^± 0.29	72.82^a ^± 0.35	0.035
BMP (%)	12.68^a ^± 0.13	12.64^a ^± 0.14	12.56^b ^± 0.17	0.010
LMP (%)	18.18^b ^± 0.15	18.14^b ^± 0.17	18.31^a ^± 0.20	0.002

	12814T>G			
		
	TT	TG	GG	
BW(g)	1534.53^b ^± 38.69	1646.77^a ^± 25.27	1538.59^b ^± 37.21	0.029
CW(g)	1356.31^b ^± 43.65	1467.70^a ^± 28.51	1358.04^b ^± 41.98	0.041
SEP (%)	86.74^a ^± 0.41	87.04^a ^± 0.26	85.53^b ^± 0.39	0.004
BMW(g)	153.03^b ^± 4.74	163.31^a ^± 3.09	150.03^b ^± 4.56	0.026
LMW(g)	220.97^b ^± 7.39	241.68^a ^± 4.83	220.35^b ^± 7.11	0.043

Note – Values are presented by the least squares means ± standard error. *Overall significance value for an effect of the genotype. The superscripts lacking a common lowercase differ significantly (*P *< 0.05). The superscripts lacking a common uppercase differ great significantly (*P *< 0.01). BW = body weight (g); CW = carcass weight (g); SEW = semi-eviscerated weight; EW = eviscerated weight; BMW = breast muscle weight (g); LMW = leg muscle weight (g); % indicates these traits relative to CW.

For 12814T>G, BW, carcass weight (CW), BMW and LMW of chickens with the TG genotype were significantly higher than those with the GG and TT genotypes, respectively (*P *< 0.05). The semi-eviscerated percentage (SEP) values of chickens with the GG genotype were significantly lower than those with the TG and TT genotypes, respectively (*P *< 0.01). No significant difference was detected for other carcass traits.

### Construction of Haplotypes and Their Frequencies

Haplotypes were constructed with the two SNPs in all 307 experimental birds by employing the PHASE program [14] of which the main function was reconstructing haplotypes from population data. Table 2 listed all four haplotypes constructed based on the two SNPs, which were accounted for 100% of all the observations. Nine diplotypes were obtained based on these four haplotypes. Among them, the frequency of the minor diplotypes was higher than 4% (Table 3).

**Table 2:** Haplotypes inferred based on the two single nucleotide polymorphisms of *CAPN3 *gene

Haplotype	11818T>A	12814T>G	Frequency (%)
H1 (TG)	T	G	22.73
H2 (TT)	T	G	18.14
H3 (AG)	A	G	32.97
H4 (AT)	A	T	26.16

**Table 3:** Diplotypes of chicken *CAPN3 *gene

Diplotype	Frequency (%)
H1H1	6.84 (21)
H1H2	12.38 (38)
H1H3	12.38 (38)
H2H2	4.24 (13)
H2H3	14.00 (43)
H2H4	8.47 (26)
H3H3	13.03 (40)
H3H4	20.52 (63)
H4H4	8.14 (25)

### Association of the Haplotypes with Carcass Traits

The mixed model analysis indicated that there were significant associations between the haplotypes and carcass traits (Table 4). Haplotypes were found to be associated with BW (g), CW (g) (both BW and CW with a *P *value smaller than 0.05), SEP (%), EP (%), BMP (%), and LMP (%) (the latter four had a *P *value smaller than 0.01). There was no significant association between the haplotypes and other traits (Table 4). The diplotype H1H2 had a significantly advantageous effect on BW, CW, and LMP (%), whereas the diplotype H2H2 was associated with SEP (%), EP (%), and BMP (%) (Table 5). Notablely, diplotype H1H3 had a negative effect on BW, CW, SEP (%), and EP (%), and H1H1 had the highest reducing effect on BMP (%) and LMP (%).

**Table 4:** Effects of haplotypes on carcass traits

Trait	*P*-value
BW (g)	0.0129
CW (g)	0.0478
SEP (%)	< 0.0001
EP (%)	0.0044
BMW(g)	0.3699
BMP (%)	< 0.0001
AFW (g)	0.5163
AFP (%)	0.4258
SFT(mm)	0.6733
LMW (g)	0.1998
LMP (%)	< 0.0001

BW = body weight (g); CW = carcass weight (g); SEW = semi-eviscerated weight; EW = eviscerated weight; BMW = breast muscle weight (g); AFW = abdominal fat weight (g); SFT = skin fat thickness (mm); LMW = leg muscle weight (g); % indicates these traits relative to CW (g).

**Table 5:** Associations between the diplotypes and chicken carcass traits

	Trait					
	
Diplotype	BW (g) *	CW (g) *	SEP (%) **	EP (%) **	BMP (%) **	LMP (%) **
H1H1	1525.81 ± 67.03	1363.02 ± 74.01	86.10 ± 0.69	72.09 ± 0.68	12.15 ± 0.34	17.72 ± 0.50
H1H2	**1655.48 ± 48.15**	**1540.09 ± 53.16**	86.89 ± 0.50	73.05 ± 0.49	12.56 ± 0.24	**18.59 ± 0.29**
H1H3	1432.47 ± 84.64	1254.29 ± 93.46	85.76 ± 0.51	71.63 ± 0.50	12.67 ± 0.25	17.86 ± 0.29
H2H2	1523.36 ± 49.22	1339.97 ± 54.35	**87.65 ± 0.87**	**73.10 ± 0.86**	**13.20 ± 0.43**	18.17 ± 0.40
H2H3	1615.98 ± 45.88	1418.61 ± 50.66	87.03 ± 0.47	72.52 ± 0.47	12.86 ± 0.23	18.45 ± 0.27
H2H4	1567.74 ± 59.06	1390.66 ± 65.22	86.08 ± 0.61	71.91 ± 0.60	12.27 ± 0.30	18.07 ± 0.35
H3H3	1600.89 ± 48.77	1423.76 ± 53.85	86.49 ± 0.51	72.62 ± 0.50	12.53 ± 0.24	18.12 ± 0.29
H3H4	1651.25 ± 37.35	1450.81 ± 41.24	87.09 ± 0.38	72.49 ± 0.38	12.57 ± 0.19	18.26 ± 0.22
H4H4	1535.84 ± 59.08	1349.38 ± 65.24	87.02 ± 0.61	73.08 ± 0.60	13.10 ± 0.30	18.06 ± 0.35

Note – Values are presented by the least squares means ± standard error. The advantageous effects of the diplotypes were marked in bold, whereas the negative effects were underlined. BW = body weight (g); CW = carcass weight (g); SEW = semi-eviscerated weight; EW = eviscerated weight; LMW = leg muscle weight (g); BMW = breast muscle weight (g); % indicates these traits relative to CW.

* P ≤ 0.05.

** P ≤ 0.01.

## Discussion

Understanding the genetic basis of protein metabolism in chickens will provide an opportunity for genetic improvement of the muscle growth traits. The study of candidate genes, based on the related results that were well characterized in humans and mice, is one of the primary methods to determine whether certain specific genes are associated to economic traits in farm animals.

Previous studies showed that calpain gene expression was correlated with muscle organization [15-17], functionally related to titin muscle [18]. In poultry, Maeda et al. [19] measured muscle protein turnover rate and calpain activity in the muscle of two quail lines divergently selected for body size. Zhang et al. [20] found that three variants in *CAPN1 *gene are associated with some carcass traits in chickens. Muscle protein turnover rate and calpain activity were significantly higher in line with small body size compared with the large line. In the current study, we analyzed *CAPN3 *gene polymorphisms and found 2 SNPs (11818T>A and 12814T>G), which showing associations (*P *< 0.05) with partial carcass traits. The result suggests that *CAPN3 *may act as a candidate gene of quantitative trait loci (QTL) in regulating muscle growth.

Haplotype or haplotype block analysis provided a practical solution to resolve the innate problems of the single-marker analysis, such as noisy, unsatisfied, and obscured important localization information [21]. Both haplotype diversity and the method of SNP selection based on maximum haplotype diversity were always preferred [22]. In this study, haplotypes were constructed using the two *CAPN3 *SNPs and were employed to evaluate the potential association between haplotypes and carcass traits. The diplotype H2H2 was found to be associated with higher SEP (%), EP (%), and BMP (%) than other diplotypes, respectively. Chickens with diplotype H1H2 had higher BW, CW, and LMP (%) than those chickens harboring other diplotypes. Apparently, haplotype H2 may be the most advantageous haplotype for carcass traits. Note that our haplotype blocks were based on two SNPs and more tag SNPs should be typed in order to detect the complete haplotype blocks in different chicken breeds.

It should be mentioned that the four native chicken breeds (HH, QY, SD, and CK) and the five commercial pure lines have been subjected to intensive artificial selection on commercial traits, such as carcass weight, eviscerated weight, and breast muscle weight, which might account for the significant deviation from the Hardy – Weinberg equilibrium for variant 11818T>A in some populations. Moreover, it is hard to include a term in the statistical analysis to adjust for relatedness among the individual chickens from the population. Our model also did not include the effects of hatch, location (e.g. cage), and rearing phase (e.g. randomization of breeds to location) that have major effects on growth traits. Therefore, different chicken populations with various domestication background and selection history, further optimized analysis model, more tag SNPs are needed to verify the genetic effects of the *CAPN3 *gene.

## Conclusion

In summary, two SNPs in intron 8 and exon 10 of *CAPN3 *gene were found in a large cohort of chicken samples from China and there was an association between *CAPN3 *haplotypes and carcass traits. It is suggestive that *CAPN3 *may be a potential major gene or in close linkage disequilibrium with a QTL for muscle growth in chicken. Further analyses of the effects of *CAPN3 *polymorphisms are essential to confirm the association between the alleles or haplotypes and chicken carcass traits.

## Methods

### Resource Populations

In total, 307 meat-type quality chicken (with a male/female ratio as 1:1) were used. These samples included 5 commercial pure lines and 4 Chinese domestic chicken breeds. All populations were raised under the same condition and were randomly selected. All the pure lines were officially recognized and were originated from the local breeds in Sichuan (CK and SD) and Guangdong (HH and QY) Provinces. The CK and SD chickens have spotty feathers, black or yellow skin, and a favorable meat quality. The HH and QY chickens are named by their yellow plumage, skin, and shank and have a high quality of meat. Different breeds were hatched in different incubators under the same condition on June 25, 2007. The birds were kept in single breed pens and slaughtered on October 13, 2007. All birds had free access to food and water. Commercial corn-soybean diets that met all NRC requirements were provided in the study. From birth to 3 week (wk) of age, birds received a starter feed (2.90 megacalories (Mcal) of metabolizable energy (ME)/kg and 20.5 g/kg of crude protein (CP)). Birds were fed a grower diet (3.00 Mcal of ME/kg and 18.5 g/kg of CP) from 4 to 6 wk of age and were transferred to the growing pens at the age of 7 wk. Before slaughter, blood was collected and the genomic DNA was isolated by the standard phenol/chloroform method. For each population, 27 to 62 chickens were randomly sampled for collecting blood and slaughtering.

### Phenotypic Measurements

Birds were raised in cage according to the conventional program of commercial broiler. Traits were measured at 90 days age, including BW of the live birds after 12 h with no access to feed, CW, semi-eviscerated weight (SEW), eviscerated weight (EW), BMW, LMW, abdominal fat weight (AFW), subcutaneous fat thickness (SFT). The CW was measured on the chilled carcass removed feathers. Semi-eviscerated weight was measured on the carcass removed trachea, esophagus, gastrointestinal tract, spleen, pancreas, and gonad. Eviscerated weight was measured on the semi-eviscerated weight after removal of head, claws, heart, liver, gizzard, glandular stomach, and abdominal fat. Subcutaneous fat thickness was measured at the caudal spondyle including the skin and fat width with a vernier caliper after dressing. The ratios of each of the above traits to CW were calculated as eviscerated percentage (EP), semi-eviscerated percentage (SEP), breast muscle percentage (BMP), and leg muscle percentage (LMP), respectively.

### PCR-SSCP Screening for CAPN3 Mutations

Nine primer pairs were designed to amplify *CAPN3 *exons according to the sequence of *Gallus gallus CAPN3 *(GenBank Accession No: NC_006092.2, GI: 118136304) (Additional file 2). Primers synthesis was completed by the TaKaRa Biotechnology Co., Ltd (Dalian, China). The PCR reaction was performed with the following condition: one denaturation cycle at 94°C for 6 min, followed by 35 cycles at 94°C for 45 s, 52°C for 45 s, 72°C for 1 min, and ended with an extension cycle at 72°C for 8 min. The 10 μL reaction volume included 0.8 μL DNA template (50 ng/μL), 5 μL 2 × Taq PCR MasterMix (Beijing TIAN WEI Biology Technique Corporation, Beijing, China), 3.6 μL ddH_2_O, 0.3 μL of each primer (10 pmol/μL). PCR products were separated on 1% agarose gel and were visualized on gel imaging system (Gel DocTMEQ170-8060) and photographed. 3 μL PCR products was denatured at 98°C for 10 min, then were placed on ice for 5 min. Electrophoresis was run at 10 V/cm on a 12% polyacrylamide gel electrophoresis for 9 to 10 h at 20°C. Gels were stained using the silver staining. Individual single strand conformation polymorphism banding pattern was determined under visible light. Samples showing different bands in the gel were further amplified and purified, and were sequenced by a commercial sequencing company (Shanghai Yingjun Biology Technique Corporation, Shanghai, China).

### Statistical Analysis

Data were analyzed using the GLM (General Linear Models Procedures) of SAS 8.0 (SAS Institute Inc., Cary, NC). The genetic effects were analyzed by a general mixed procedure in the SAS package, and the following model was used:

Y = μ + B + S + G + bX + e

where Y = the dependant variable; μ = the population mean; B = fixed effect of the breed; S = fixed effect of the sex; G = genotype value; e = random effect; b = the regression coefficients of the linear regressions on BW (X); BW was included in the model as it was significant for traits (SEW, EW, BMW, LMW, and AFW) at single locus. Multiple comparisons were analyzed with the least square means (LSM), and it was excluded from the model if its effect had a *P *value larger than 0.05 on a given trait. The significance of the least square means was tested with the Duncan's Multiple Range test (*P *< 0.01). The data for all carcass traits were verified for normal distribution by using the Shapiro-Wilks test in SAS 8.0. None of them were normally distributed. Thus, the parameters of BW, CW, SEW, EW, BMW, LMW, AFW, and SFT were estimated on the square root scale before they were analyzed.

### Haplotype Construction

Haplotypes were constructed based on the 2 SNPs identified in all 307 experimental animals by using the PHASE 2.0 program [14]. The program reconstructed haplotypes from a population data. The genetic status of the subjects was expressed as the combination of two haplotypes (diplotype configuration). Genetic effects of the haplotypes were performed with the mixed model mentioned above.

## Authors' contributions

ZRZ carried out the genotyping work, managed the data, statistical analyses, interpretation of the data and drafted the manuscript. YPL and QZ conceived the study and the design, initiated its key collaborations. HRD and XSJ collected the field data. YGY conceived the design and revised the manuscript. All authors read and approved the final manuscript.

## Supplementary Material

Additional file 1 **Supplemental table 1.** Genotype and allele frequency of the CAPN3 SNPs in chicken populations. Click here for file

Additional file 2 **Supplemental table 2.** Primers for screening the single nucleotide polymorphisms in chicken CAPN3 gene. Click here for file
