# Supplementary material for: Identification and association of the single nucleotide polymorphisms in calpain3 (CAPN3) gene with carcass traits in chickens
Source: BMC Genet. 2009 Mar 5;10:10. doi: 10.1186/1471-2156-10-10 (PMC2656522; doi:10.1186/1471-2156-10-10)
Supplement: Additional file 1 — Supplemental table 1. Genotype and allele frequency of the CAPN3 SNPs in chicken populations. [file 1471-2156-10-10-S1.doc]

Genotype and allele frequency of the *CAPN3* SNPs in chicken populations.

| Line | No | 11818T>A | | | | | | | 12814T>G | | | | | | |
| --- | --- | --- | --- | --- | --- | --- | --- | --- | --- | --- | --- | --- | --- | --- | --- |
|  |  | AA | AT | TT | A | T | *P*-value ^a^ | *P*-value ^b^ | TT | TG | GG | T | G | *P*-value ^a^ | *P*-value ^b^ |
| S03 | 30 | 11 (0.37) | 10 (0.33) | 9 (0.30) | 0.53 | 0.47 | 0.07 | 0.7247 | 16 (0.53) | 9 (0.30) | 5 (0.17) | 0.68 | 0.34 | 0.09 | *<*0.001 |
| S01 | 30 | 10 (0.33) | 15 (0.50) | 5 (0.17) | 0.58 | 0.42 | 0.87 |  | 7 (0.23) | 15 (0.50) | 8 (0.27) | 0.48 | 0.52 | 0.99 |  |
| D99 | 30 | 12 (0.40) | 9 (0.30) | 9 (0.30) | 0.55 | 0.45 | 0.03 |  | 3 (0.10) | 11 (0.37) | 16 (0.53) | 0.28 | 0.72 | 0.59 |  |
| S02 | 30 | 13 (0.43) | 10 (0.34) | 7 (0.23) | 0.60 | 0.40 | 0.09 |  | 7 (0.23) | 20 (0.67) | 3 (0.10) | 0.57 | 0.43 | 0.05 |  |
| S05 | 30 | 15 (0.50) | 8 (0.27) | 7 (0.23) | 0.63 | 0.37 | 0.02 |  | 6 (0.20) | 10 (0.33) | 14 (0.47) | 0.37 | 0.63 | 0.12 |  |
| QY | 33 | 10 (0.31) | 15 (0.45) | 8 (0.24) | 0.53 | 0.47 | 0.61 |  | 5 (0.15) | 22 (0.67) | 6 (0.18) | 0.48 | 0.52 | 0.05 |  |
| HH | 35 | 12 (0.34) | 16 (0.46) | 7 (0.20) | 0.57 | 0.43 | 0.69 |  | 4 (0.11) | 22 (0.63) | 9 (0.26) | 0.43 | 0.57 | 0.09 |  |
| SD | 62 | 28 (0.45) | 21 (0.34) | 13 (0.21) | 0.62 | 0.38 | 0.03 |  | 5 (0.08) | 21 (0.34) | 36 (0.58) | 0.25 | 0.75 | 0.45 |  |
| CK | 27 | 15 (0.56) | 6 (0.22) | 6 (0.22) | 0.67 | 0.33 | 0.01 |  | 8 (0.30) | 11 (0.40) | 8 (0.30) | 0.50 | 0.50 | 0.34 |  |

Note - Commercial pure lines - S01, S02, S03, S05, and D99; HH – Huiyang Huxu chicken; QY – Qingyuan Ma chicken; CK – Caoke chicken; SD – Shandi chicken. ^a^ *P*-value is the probability of the χ^2^-test for the Hardy-Weinberg equilibrium. ^b^ *P*-value is the probability of the Chi-square test for genotype frequency
