# Supplementary material for: Identification and association of the single nucleotide polymorphisms in calpain3 (CAPN3) gene with carcass traits in chickens
Source: BMC Genet. 2009 Mar 5;10:10. doi: 10.1186/1471-2156-10-10 (PMC2656522; doi:10.1186/1471-2156-10-10)
Supplement: Additional file 2 — Supplemental table 2. Primers for screening the single nucleotide polymorphisms in chicken CAPN3 gene. [file 1471-2156-10-10-S2.doc]

**Additional file 2:**

Primers for screening the single nucleotide polymorphisms in chicken *CAPN3* gene.

| Primer number | Forward Primer  (5’—3’) | Reverse Primer  (5’—3’) | Annealing temperature (°C) | Product length (bp) | Exon coverage |
| --- | --- | --- | --- | --- | --- |
| 1 | GCTTGCTTACAGGCGATT | ATAATTACATCCCCTTAATGC | 52 | 169 | 3 |
| 2 | TTCCCTGCACACAGTGAACCC | CCAAAGAGAGCATGACACGG | 56 | 221 | 4 |
| 3 | TTCCTCTTTCCAAGACTCCA | TTCTGCCATTTCTTATGACTC | 52 | 229 | 5 |
| 4 | TGCCTTCTCTTTCAATTAGAC | AAACCTGATACCTCGTTGT | 52 | 222 | 8 |
| 5 | CACTAACGAGGCTTACAGTA | GTGACCCAGTTTTGTTACAGT | 51 | 233 | 9 |
| 6 | TTCAATTCTTCCAGGATATCA | TGGTATCTCTTAAGACCCATT | 52 | 202 | 10 |
| 7 | AATGGACATCCCCAGATACTT | CTGGCCAGACCAAGCTCTAT | 53 | 227 | 16 |
| 8 | CCTTCATTTTCCTGGATCATA | TCTGTGAAGAAGTTGGGTTAG | 53 | 197 | 17 |
| 9 | GAGGAAAAAGTGATTGCTGAC | GGGGAGTAAAACTCAGGT | 52 | 180 | 19 |
